# Decoding exonic intron retention from sequence: an exploratory study in the human genome

**DOI:** 10.3389/fbinf.2026.1801042

**Published:** 2026-05-20

**Authors:** Luca Faretra, Francesco Napolitano, Massimo Pancione, Luigi Cerulo

**Affiliations:** 1 Bioinformatics Lab–Department of Science and Technology, University of Sannio, Benevento, Italy; 2 Biogem scarl, Research Institute, Ariano Irpino (AV), Italy

**Keywords:** alternative splicing, feature selection, intron retention, machine learning, rna sequence

## Abstract

Alternative splicing enables the production of multiple transcripts from a single gene. Among its major forms, intron retention is particularly well characterised in plants, but with ample evidence also in animals. While most introns are constitutively spliced to ensure efficient removal from protein-coding gene transcripts, a subset exhibits an intrinsic sequence-level predisposition to be selectively retained under specific cellular or environmental conditions. However, the synergistic interplay of sequence features that creates this intrinsic predisposition remains only partially understood. Here, we present an exploratory, sequence-centric analysis of intron retention in the human genome. We systematically extract exon–intron–exon units from high-confidence transcript annotations and characterize each event using a broad set of sequence-derived features, including intron and exon architecture, splice site strength, nucleotide composition, transposable element content, and RNA-binding protein (RBP) binding patterns. Using integrative Random Forest and LASSO frameworks, we evaluate both the individual and joint discriminative power of these features. Our results show that intrinsic predisposition to intron retention cannot be explained by any single determinant, but instead emerges from the synergistic interplay of multiple factors. Intron length and GC content represent dominant predictors, while splice site strength, specific transposable element families, and structured RBP binding signatures provide additional explanatory power. Notably, RBP-derived features alone retain substantial predictive capacity, highlighting the importance of regulatory signal organization beyond basic sequence composition. Overall, this study provides a unified view of the sequence-level determinants underlying intron retention in humans and establishes a scalable computational framework for a deeper understanding of post-transcriptional gene regulation and its potential dysregulation.

## 1 Introduction

Splicing is a critical step in eukaryotic gene expression, enabling the precise removal of introns from pre-mRNA to produce mature coding transcripts. Beyond constitutive splicing, the splicing machinery frequently engages in alternative splicing (AS), generating multiple mRNA isoforms from a single gene. This process substantially expands the functional diversity of both the transcriptome and the proteome. AS is known to play a fundamental role in development, stress responses, and disease (Tao et al., 2024).

Among the various forms of AS, intron retention has been recognized as a dominant splicing mode in plants (Kim et al., 2007), and in recent years it has gained increasing attention for its functional relevance also in animal systems (Middleton et al., 2017). Intron retention occurs when an intron flanked by two exons is not excised by the splicing machinery and is instead retained within the transcript, giving rise to an exonic retained intron (Tao et al., 2024). In annotated transcriptomes, such events are defined by the coexistence of transcript isoforms in which the same intron is either spliced out or retained, reflecting an intrinsic, sequence-encoded potential for AS. In addition to these annotation-defined events, intron retention can also be observed in a broader, context-dependent manner through transcriptome-wide RNA sequencing analyses, where introns show differential inclusion across cell types, developmental stages, or experimental conditions. Recent large-scale studies (Park and Xing, 2025) have demonstrated that intron retention is a genetically regulated process, with thousands of specific genetic variants (irQTLs) directly influencing splicing outcomes across diverse human tissues. Notably, a significant fraction of these variants is associated with complex phenotypic traits and disease-related loci identified in GWAS, highlighting the clinical relevance of IR. Such events, commonly detected by tools such as rMATS (Shen et al., 2014), MAJIQ (Vaquero-Garcia et al., 2016) and related methods, capture dynamic regulatory responses and may involve heterogeneous mechanisms, including partial intron inclusion or alternative splice site usage.

While the vast majority of introns are constitutively spliced, retained introns can be detected in the nucleus and, in several cases, also in the cytoplasm. Intron retention can prevent nuclear RNA export, leading to the degradation or detention of transcripts within the nucleus. These transcripts can form a nuclear pool of partially spliced RNA, acting as a reservoir that feeds the cytoplasmic mRNA pool once splicing is completed. Alternatively, some intron-containing transcripts are exported to the cytoplasm as alternative mRNA isoforms; these may encode alternative proteins or undergo altered translation and decay (Yeom et al., 2021).

In this study, we focus specifically on protein coding exonic retained introns, with the aim of characterizing the sequence-level features that predispose an intron to retention independently of cellular context. Beyond the intrinsic signals that predispose an intron to be retained, other regulatory layers determine the actual splicing outcome under different cellular or environmental conditions. Recent integrative analyzes have highlighted that the chromatin architecture plays a dominant role in this dynamic regulation. Specifically, increased chromatin accessibility and specific nucleosome positioning act as a molecular guide for intron retention (Petrova et al., 2022). So, while sequence features provide the necessary foundation, the permissive state of chromatin—characterized by nucleosome-free regions—facilitates the acceleration of RNA Polymerase II, thereby promoting retention. Furthermore, the involvement of the chromatin state has recently been explored through large-scale deep learning frameworks (Daoud and Ben-Hur, 2025). Models have shown that retention-susceptible regions are strongly associated with specific transcription factor (TF) binding patterns and histone modifications. This indicates that a vast network of TFs (such as MYC and CTCF) contributes to the establishment of the chromatin environment necessary for co-transcriptional splicing regulation. These findings suggest that the splicing code is not merely a collection of isolated motifs, but a complex coordination between the primary sequence and the epigenetic landscape.

Several studies have investigated the factors that predispose the intron retention, identifying intron length as a critical determinant of splicing outcomes (Farlow et al., 2012; Guo et al., 1993). A well-known example is provided by the second intron of the white gene in *Drosophila melanogaster*. In nuclear extracts from human HeLa cells, the native 74-nucleotide (nt) intron is inefficiently spliced, whereas increasing its length to 84–90 nt markedly enhances splicing efficiency. In contrast, in nuclear extracts from *Drosophila* Kc cells, the 74 nt intron is efficiently spliced, while extending it beyond the 84–90 nt threshold leads to a dramatic reduction in splicing efficiency (Guo et al., 1993).

Splice site strength, typically quantified using consensus-based scoring schemes such as position weight matrices or maximum entropy models, represents another critical determinant of splicing regulation. This factor was specifically investigated in bovine growth hormone pre-mRNA, where intron D retention was analyzed through site-directed mutagenesis and expression in CHO cells (Dirksen et al., 1995). Notably, substitution of the native 5′ and 3′ splice sites with stronger consensus sequences completely abolished intron retention, resulting in constitutive splicing. Conversely, further weakening of the 3′ splice site, particularly within the polypyrimidine tract, led to a drastic reduction in overall splicing efficiency.

Another player in determining splicing events is the intronic GC content. Elevated GC levels can promote the formation of stable RNA secondary structures that physically hinder access to splice sites (Sznajder et al., 2018). In addition, high GC content has been associated with a reduced RNA polymerase II elongation rate; this kinetic slowdown can favor intron retention by altering the temporal coordination between transcription and spliceosome assembly (Veloso et al., 2014).

Transposable elements (TEs), mobile DNA sequences capable of inserting into diverse genomic regions, also appear to influence splicing regulation. In several plant species, TEs are enriched within retained introns, particularly in proximity to the 3′ splice site. This enrichment may reduce local RNA structural flexibility and thereby contribute to intron retention (Li et al., 2014). Beyond plant systems, TEs—particularly SINE retrotransposons, such as ID elements—are also functional in the mammalian nervous system (Buckley et al., 2011). These intronic sequences are often retained to act as cis-acting signals for the dendritic targeting of mRNAs. Such evidence suggests that the co-optation of repetitive sequences is a conserved regulatory strategy, linking intron retention directly to the spatial control of protein function.

Binding sites for RNA-binding proteins (RBPs) constitute an additional layer of post-transcriptional regulation that includes the control of splicing. Beyond the core spliceosomal machinery—comprising the small nuclear ribonucleoproteins U1, U2, U4, U5, and U6—splicing outcomes can be fine-tuned by cis-regulatory sequence motifs that recruit accessory splicing factors. These motifs are classified according to their genomic location and functional effect as exonic or intronic splicing enhancers or silencers (ESEs, ESSs, ISEs, and ISSs) (McManus and Graveley, 2011). They mediate the binding of a wide range of RBPs, including members of the serine/arginine-rich (SR) protein family. Notably, retained introns exhibit a higher density of SR protein binding sites, and depletion of these proteins—either through knockdown experiments or disease-associated dysfunction—has been shown to impair splicing efficiency and increase intron retention levels (Middleton et al., 2017).

Numerous studies have examined sequence and regulatory features associated with intron retention across different organisms and biological contexts. Large-scale transcriptomic analyses have primarily identified retained introns based on expression patterns inferred from RNA-seq data, revealing their widespread occurrence and functional relevance (Braunschweig et al., 2014; Middleton et al., 2017). While effective for detecting intron retention events, these approaches inherently depend on the specific cellular or environmental conditions under study, making it difficult to disentangle intrinsic sequence-driven determinants from context-dependent regulatory effects.

Complementary investigations have focused on specific introns or limited sets of genes, often through targeted experimental systems or comparative analyses (Guo et al., 1993; Dirksen et al., 1995; Sakabe and De Souza, 2007). These studies have provided important mechanistic insights into how individual features—such as intron length or splice site composition—influence splicing outcomes. However, their restricted scope limits the extent to which their conclusions can be generalized to transcriptome-wide intron retention mechanisms.

At a broader level, multiple sequence features, including intron length, splice site strength, GC content, RNA secondary structure, transposable element insertions, and RNA-binding protein motifs, have been independently associated with intron retention (Sakabe and De Souza, 2007; McManus and Graveley, 2011; Li et al., 2014). Nevertheless, most studies have considered these features in isolation, with limited efforts to quantify their relative contributions while accounting for confounding effects or to investigate how they act in combination. As a result, despite comprehensive reviews highlighting the multifactorial nature of intron retention (Monteuuis et al., 2019; Porras-Tobias et al., 2025), the extent to which intron fate is governed by synergistic interactions among sequence features remains insufficiently explored.

To address these limitations, we present a systematic, sequence-centric analysis of intron retention in the human genome. We curated a comprehensive set of retained introns (RIs) and constitutively spliced introns (CIs) from high-confidence transcript annotations and characterized each event using a broad panel of sequence-derived features encompassing intron and exon architecture, splice site strength, nucleotide composition, repetitive elements, and RNA-binding protein (RBP) binding patterns.

Rather than evaluating these features in isolation, we adopt an integrative exploratory framework that explicitly models their joint contribution to intron fate. Using a Random Forest–based classification strategy, we quantify the collective discriminative power of heterogeneous sequence features and assess their relative importance while accounting for strong class imbalance and potential confounding effects such as intron length. This approach enables us to identify dominant and synergistic sequence-level determinants of intron retention, providing a more unified view of the regulatory mechanisms underlying this splicing outcome.

## 2 Data and methods

### 2.1 Dataset generation

We initiated the analysis using transcript annotations from GENCODE release 49 (GRCh38. p14). To ensure the inclusion of high-confidence splicing events, the analysis was restricted to transcripts mapped to the primary assembly (chromosomes 1–22, X, and Y) and to NM RefSeq transcripts, which are manually curated. Alternative haplotypes, unplaced and unlocalized contigs, as well as annotated pseudogenes, were excluded.

In total, we collected 69,633 transcripts corresponding to 19,396 genes. Transcripts were decomposed into exon–intron–exon (E–I–E) units. To retain only unambiguous splicing contexts and to avoid confounding effects arising from alternative splice site usage or complex transcript architectures, we considered only E–I–E units exhibiting consistent splice site usage across all transcripts in which they occurred.

E–I–E units in which both flanking exons and the intervening intron were spliced identically across all annotated transcripts were classified as constitutively spliced introns (CIs). Conversely, E–I–E units were classified as retained introns (RIs) when the intron was spliced out in a subset of transcripts but retained in others, provided that the 5′ splice site of the upstream exon and the 3′ splice site of the downstream exon were identical and invariant across both spliced and retained isoforms.

To enable robust downstream sequence-based analyses, E–I–E units were further filtered to exclude exons ≤ 6 nt, introns ≤ 26 nt, and events containing ambiguous nucleotides (N). After all filtering steps, a total of 144,241 CIs and 628 RIs were retained for subsequent analyses.

### 2.2 Extraction of sequence features

We extracted a total of 2,397 features from the nucleotide sequences of the identified exon–intron–exon (E–I–E) units. As summarized in Table 1, these features were organized into five groups and included: (i) upstream exon, intron, and downstream exon lengths and GC content; (ii) splice site strength, computed using the MaxEntScan algorithm (Yeo and Burge, 2003); (iii) coverage fractions of repetitive element subfamilies derived from RepeatMasker annotations (Tempel, 2012); and (iv) RNA-binding protein (RBP) binding site features, represented through a wavelet-based decomposition of the binding signal.

**TABLE 1:** Summary of the features extracted for each exon-intron-exon event.

Feature	Description	Number
Lengths	Genomic lengths of the intron and its flanking exons	3
GC content	GC content frequencies for intron and flanking exon regions	3
Splice sites strength	Donor and acceptor splice site strengths calculated via MaxEntScan algorithm	2
Repetitive elements	Fractional coverage of specific repetitive element subfamilies within introns	49
RBP binding sites	Multi-scale wavelet descriptors of RBP binding energy, sparsity, and spatial distribution	2340

Genomic coordinates of repetitive elements were obtained from the RepeatMasker UCSC track. To ensure high-confidence annotations, repetitive elements with uncertain classifications—indicated by “?” or annotated as “unknown” in the repClass or repFamily fields—were excluded. Overlaps between intronic regions and repetitive elements were computed using the findOverlaps function from the GenomicRanges package (Lawrence et al., 2013). For each intron and each repeat subfamily, the overlap was quantified as the proportion of the intronic length covered by elements belonging to that subfamily, resulting in a normalized coverage fraction independent of intron length.

RNA-binding protein (RBP) binding sites were identified using position weight matrices (PWMs) obtained from the ORNAMENT database (Benoit Bouvrette et al., 2020). To ensure biological specificity, the analysis was restricted to 130 human-derived RBP PWMs. Following the authors’ methodology, binding thresholds were empirically determined for each PWM as the 50th percentile of the cumulative score distribution computed across all possible 7-mer sequences.

For each PWM, nucleotide sequences were scanned and represented as a binary step signal

$$x={\left\{x_{i}\right\}}_{i=1}^{N},\;x_{i}\in \left\{0,1\right\},$$

where $x_{i}=1$ indicates a position exceeding the PWM-specific threshold and $x_{i}=0$ otherwise. For RBPs characterized by multiple PWMs, the corresponding binary signals were merged into a single representative signal using a position-wise logical OR operation.

To characterize the multiscale statistical organization of RBP binding sites, each binary signal was decomposed using a discrete wavelet transform (DWT) (Percival and Walden, 2000). Specifically, we applied a Maximum Overlap Discrete Wavelet Transform (MODWT) with the Haar wavelet as the filter. Unlike the standard decimated DWT, MODWT is translation invariant and does not require signal length to be a power of two, making it particularly well suited for genomic sequences of variable length and for capturing spatial patterns independently of their absolute position along the sequence.

Each signal was decomposed into six levels (L=1,…,6) , producing a set of wavelet coefficient vectors ${\left\{w^{\left(l\right)}\right\}}_{l=1}^{6}$ . For each decomposition level l , the following three descriptors were computed:

- **Relative Wavelet Energy**, defined as
$$E_{l}=\frac{\sum_{i}{\left(w_{i}^{\left(l\right)}\right)}^{2}}{\sum_{k=1}^{6}\sum_{i}{\left(w_{i}^{\left(k\right)}\right)}^{2}},$$
  which quantifies the relative contribution of scale l to the total signal energy.
- **Fraction of Outliers**, defined as
$$O_{l}=\frac{1}{\left|w^{\left(l\right)}\right|}\underset{i}{\sum }I\left(|w_{i}^{\left(l\right)}|>M_{l}+3\cdot MAD_{l}\right),$$
  where $M_{l}$ and $MAD_{l}$ denote the median and median absolute deviation of $w^{\left(l\right)}$ , respectively, and I(⋅) is the indicator function. This descriptor captures localized structural discontinuities in the binding signal.
- **Sparsity Proxy**, defined as
$$S_{l}=\frac{\|w^{\left(l\right)}{\|}_{1}}{\|w^{\left(l\right)}{\|}_{2}},$$
  which characterizes the concentration of the signal at scale l , with lower values indicating sparser representations.

From a computational standpoint, the MODWT decomposition has linear time complexity with respect to sequence length, O(N⋅L) , where N is the length of the sequence and L the number of decomposition levels. Given the moderate sequence lengths and the fixed number of levels used in this study, the overall computational cost of feature extraction remained tractable and scalable to genome-wide analyses.

### 2.3 Model building

We employed a Random Forest classifier implemented in the ranger R package (Wright and Ziegler, 2017) to evaluate the predictive performance of the extracted features and to quantify their relative importance under different modeling settings. To address the pronounced class imbalance between retained introns (RIs; n=628 ) and constitutively spliced introns (CIs; n=144,241 ), we adopted a cost-sensitive learning strategy. Class weights were defined as the ratio between the majority and minority class sizes, thereby assigning a higher misclassification penalty to RIs.

Given the high dimensionality of the predictor space (2,397 features), models were trained using 1,000 trees with an mtry parameter set to 100, corresponding to approximately 4% of the available variables. This choice increases the probability that informative but correlated features are jointly considered at split nodes, which is particularly relevant in the presence of structured and partially redundant genomic features.

Model performance was assessed using a 5-fold cross-validation scheme and was evaluated using the area under the receiver operating characteristic curve (AUC-ROC). Feature relevance was quantified using permutation-based variable importance measures. To ensure robust comparisons and account for potential variability across models, raw importance scores were standardized by twice their standard deviation (2σ) and subsequently transformed using an inverse hyperbolic sine (asinh) function. This approach provides a pseudo-logarithmic scale that remains defined for zero or negative values—which may arise during the permutation of non-informative features—while effectively compressing the range of high-magnitude predictors to facilitate visualization and interpretation.

To control for potential biases arising from differences in sequence length, an additional model was trained following covariate matching on the training data. A length-matched subset was constructed using Mahalanobis distance–based nearest-neighbor matching implemented in the MatchIt package (Ho et al., 2011). Matching was performed on the joint distribution of upstream exon, intron, and downstream exon lengths using a 20:1 matching ratio. This procedure ensured that the distributions of length-related variables were statistically indistinguishable between RIs and CIs, enabling assessment of the predictive contribution of other sequence features independently of length.

Finally, to isolate the specific regulatory contribution of RNA-binding proteins, a third Random Forest model was trained using only RBP binding site–derived features as predictors. Comparison of performance across models allowed us to quantify the extent to which RBP-related features alone contribute to intron retention prediction when structural genomic features are excluded.

To further validate the robustness of our results and address potential multicollinearity among correlated sequence features (e.g., the relationship between intron length and RBP binding density), we implemented an additional feature selection step using the LASSO (Least Absolute Shrinkage and Selection Operator) algorithm. Unlike Random Forest, which captures non-linear synergies by considering multiple variables at each split node, LASSO applies a linear penalty that tends to collapse redundancy by selecting a single representative predictor among a group of correlated variables. This complementary approach allowed us to determine whether the predictive signal of a global feature, such as intron length, could be effectively represented by more granular regulatory signatures, such as the multiscale organization of RBP binding sites. Furthermore, to ensure the inclusion of only the most impactful predictors, we applied a conservative threshold, removing any features with an absolute value of the LASSO coefficient lower than $10^{-5}$ . Next, we performed a comprehensive correlation analysis to characterize the regulatory context of the identified predictors. Specifically, we calculated the Pearson correlation coefficient between each LASSO-selected feature and all other variables in the original feature matrix to evaluate whether the selected signatures acted as independent drivers or as proxies for broader genomic patterns.

## 3 Results

### 3.1 Sequence architecture distinguishes retained and constitutively spliced introns

**FIGURE 1:**
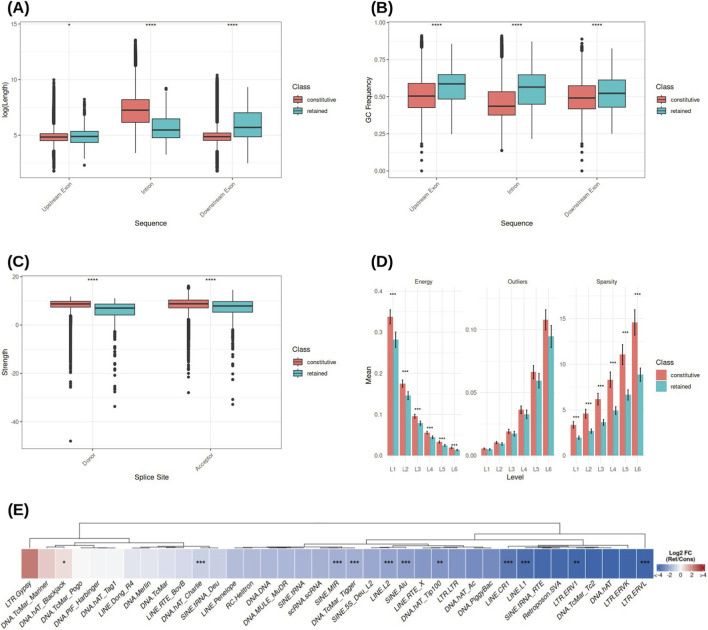
Exploratory analysis of features distinguishing retained introns from constitutively spliced ones. **(A)** Distribution of genomic lengths, **(B)** GC content, **(C)** donor and acceptor splice site strengths, **(D)** RBP binding factors descriptors at different levels, and **(E)** differences in repetitive elements content.

Exploratory analyses revealed pronounced differences between retained introns (RIs) and constitutively spliced introns (CIs) in both sequence architecture and nucleotide composition. RIs are significantly shorter than CIs (p<0.0001) , whereas their downstream exons are significantly longer (p<0.0001) , as shown in Figure 1A. In addition, RIs exhibit a markedly higher GC content compared to CIs (p<0.0001) , a trend that also extends to their flanking exonic regions ( p<0.0001 ; Figure 1B). Both results are confirmed in (Braunschweig et al., 2014).

Analysis of splice site strength using MaxEntScan further revealed that RIs are characterized by weaker donor and acceptor sites relative to CIs ( p<0.0001 ; Figure 1C), indicating a reduced intrinsic affinity for spliceosome recognition.

### 3.2 Distinct RBP binding signal organization characterizes intron fate

Wavelet-based analysis of RNA-binding protein (RBP) binding signals uncovered distinct organizational patterns differentiating RIs from CIs. As shown in Figure 1D, CIs display higher relative wavelet energy across all decomposition levels compared to RIs (p<0.001) , consistent with a higher overall density of RBP binding motifs. Moreover, CI signals exhibit significantly higher sparsity values (p<0.001) , particularly at coarser scales (levels 4–6), indicating a more heterogeneous and clustered distribution of binding sites. In contrast, RI signals tend to be more uniform and less structured. Higher sparsity values indicate a more clustered and heterogeneous organization of binding sites, consistent with localized regulatory hubs.

No significant differences were observed between RIs and CIs in outlier density. This suggests that intron retention is not driven by rare, high-intensity binding events, but rather by global differences in regulatory signal organization.

### 3.3 Retained introns are depleted of specific transposable element classes

Analysis of repetitive element content revealed that RIs are significantly depleted of several classes of transposable elements, including LINE L1 and L2, as well as SINE Alu and MIR elements ( p<0.001 for all comparisons), relative to CIs (Figure 1E). This depletion suggests that the presence of specific repetitive element architectures may be associated with efficient constitutive splicing rather than intron retention in the human genome.

### 3.4 Integrated sequence features enable accurate prediction of intron retention

**FIGURE 2:**
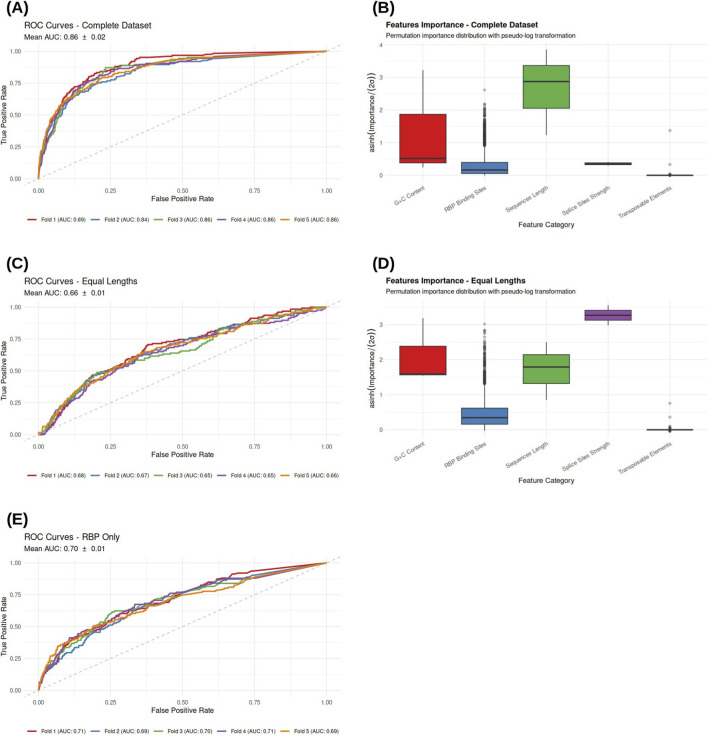
Performance and feature importance distribution of the classification models. **(A)** ROC curves showing the classification performance on the complete dataset (Mean AUC: 0.86±0.02 ). **(B)** Permutation importance distribution for the complete dataset. **(C)** ROC curves for the model trained on sequences with equal length distribution (Mean AUC: 0.66±0.01 ). **(D)** Feature importance distribution for the equal-length dataset. **(E)** ROC curves for the model using only RBP-related features (Mean AUC: 0.70±0.01 ).

The Random Forest classifier trained on the complete feature set achieved a mean AUC of 0.86±0.02 (Figure 2A), indicating strong discriminative performance between RIs and CIs. Despite the limited number of retained introns, performance estimates were stable across cross-validation folds.

**FIGURE 3:**
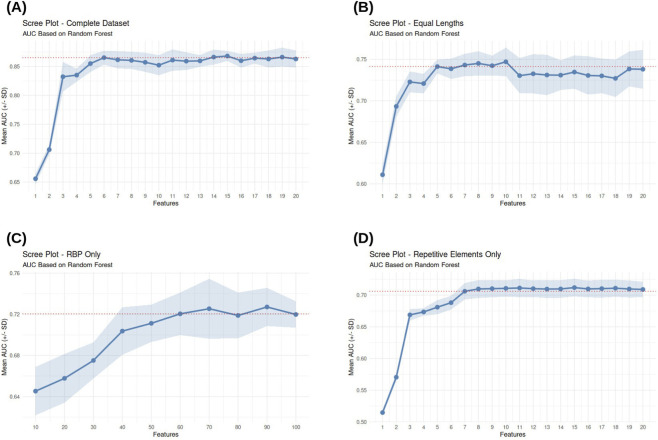
Incremental performance analysis (Scree Plot) of the Random Forest model. The mean AUC is plotted as a function of the number of features included in the model, ranked by their Permutation Importance. The four panels represent different data subsets: **(A)** Complete dataset, **(B)** Dataset balanced for intron length, **(C)** RNA-binding protein features only, and **(D)** Repetitive elements only.

Permutation-based importance analysis (Figure 2B) identified sequences length as dominant predictor. Analysis of the performance scree plot (Figure 3A) reveals that the model reaches a performance plateau with the inclusion of only 6 features (Table 2), including intron and downstream exon length, and intron GC content. Among RNA-binding protein–derived features, only a limited subset—including *MSI1* and *KHSRP* emerged as strong contributors.

**TABLE 2:** Top features obtained for each model using random forest. For clarity, only the unique RBPs associated with the top-ranked wavelet features are reported.

Complete Model	Equal Lengths Model	RBP-Only Model	Repetitive Elements Only Model
Intron length	Donor strength	MSI1	LTR ERVL MaLR
Intron GC	Intron GC	SRSF4	LINE L2
Downstream Exon length	PPRC1	HNRNPD	SINE Alu
MSI1	Acceptor strength	PCBP1	DNA TcMar tigger
KHSRP	ZNF326	KHSRP	Simple repeat
-	-	PCBP2	LINE L1
-	-	HNRNPA0	SINE MIR
-	-	ELAVL4	-
-	-	SRSF5	-
-	-	ZNF326	-
-	-	ELAVL1	-
-	-	PTBP1	-
-	-	RBM15B	-
-	-	FUBP1	-
-	-	KHDRBS3	-
-	-	KHDRBS1	-
-	-	HNRNPDL	-
-	-	hnRNPK	-
-	-	BOLL	-
-	-	KHDRBS2	-
-	-	PUM1	-
-	-	RBM4B	-

While the Random Forest model identified global structural variables as dominant, the LASSO model prioritized a specific set of RBP binding signatures; notably, 23% of the RBPs identified by the Random Forest were also independently selected by the LASSO model. In this model, the top-ranked predictors were *RBM41*, *RBFOX2*, *PUM1*, and *YB1*, followed by *ELAVL4* (Table 3). Interestingly, the length features, which ranked highest in the Random Forest permutation importance, were not selected as primary predictors by the LASSO algorithm. Instead, the model retained more granular regulatory features, including the downstream exon GC content, transposable elements (*SINE Alu*, *SINE MIR*, *LINE L1*, and *LINE L2*), and specific RBP motifs.

**TABLE 3:** Top features obtained for each model using the lasso regression. For clarity, only the unique RBPs associated with the top-ranked wavelet features are reported.

Complete Model	Equal Lengths Model	RBP-Only Model	Repetitive Elements Only Model
RBM41	Donor strength	RBFOX2	LINE L1
RBFOX2	Acceptor strength	RBM41	SINE Alu
PUM1	Downstream Exon GC	HNRNPD	SINE MIR
YB1	RBM25	IGF2BP3	LINE L2
ELAVL4	-	SF1	LINE CR1
Downstream Exon GC	-	ELAVL4	-
SF1	-	FUBP1	-
RC3H1	-	MSI1	-
HNRNPA1L2	-	RBM47	-
MSI1	-	KHSRP	-
KHSRP	-	BRUNOL6	-
SINE Alu	-	SFPQ	-
FMR1	-	HNRNPAB	-
IGF2BP3	-	pUf68	-
SINE MIR	-	PABPC5	-
NOVA1	-	TARDBP	-
RBM47	-	NOVA1	-
pUf68	-	SRSF9	-
SFPQ	-	HNRNPF	-
PABPC5	-	ILF2	-
CPEB2	-	HNRNPA1L2	-
SRSF2	-	MBNL1	-
LINE L2	-	SRSF2	-
BRUNOL6	-	ENOX1	-
MBNL1	-	ZFP36	-
IGF2BP2	-	RBM24	-
ILF2	-	hnRNPLL	-
Donor strength	-	SNRNP70	-
LINE L1	-	RBM28	-
TARDBP	-	RBM23	-
SRSF8	-	RBM25	-
Acceptor strength	-	-	-
hnRNPLL	-	-	-
LIN28A	-	-	-
SF2	-	-	-
SNRNP70	-	-	-
RBM24	-	-	-
RBM23	-	-	-
RBM28	-	-	-
HNRNPAB	-	-	-
ENOX1	-	-	-

**FIGURE 4:**
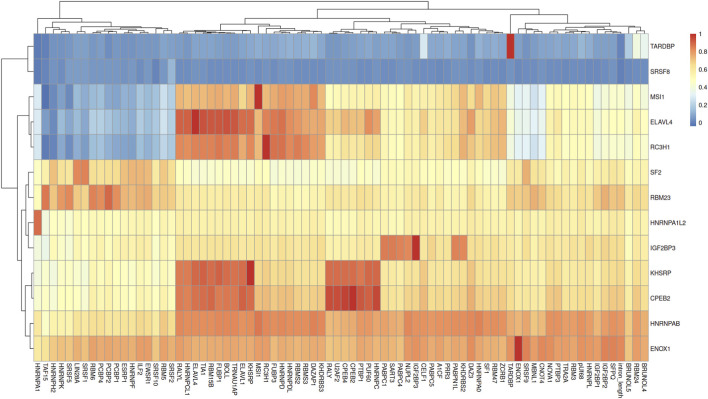
Global Correlation Landscape of LASSO-Selected Predictive Features. The heatmap displays the maximum Spearman correlation coefficients (r≥0.8) between genomic features selected by the LASSO model (Y-axis) and the broader set of functional genomic variables (X-axis).

To investigate the discrepancy, we performed a Spearman correlation analysis between the LASSO-selected features and the complete feature set (Figure 4). Several prioritized RBP signatures, such as *ENOX1* (r=0.81) and *TARDBP* (r=0.90) , showed strong positive correlations with intron length, while the majority of the selected predictors exhibited low-to-moderate associations. Notably, we observed significant inter-protein correlations, with several RBP motifs showing strong co-occurrence patterns.

Among the RBP features, consistency was observed between the two modeling approaches, with *MSI1* and *KHSRP* being identified as relevant contributors in both the Random Forest and LASSO frameworks.

### 3.5 Length-controlled models reveal secondary determinants of retention

When training the Random Forest on a length-matched dataset, predictive performance decreased to a mean AUC of 0.66±0.01 (Figure 2C), highlighting the central role of sequence length in intron fate determination.

Under these controlled conditions, the hierarchy of predictive features shifted, with splice site strength emerging as the most informative parameter (Figure 2D). Consistently, the corresponding scree plot (Figure 3B) indicates a rapid saturation of the model’s discriminative power, with the performance plateauing after the inclusion of only the top 5 features, reaching an AUC of 0.74±0.01 including the donor and acceptor strength, the intron GC content, and the RBPs *PPRC1* and *ZNF326* (Table 2).

The LASSO model also prioritized donor strength and acceptor strength as the most discriminative parameters (Table 3). Beyond core splicing signals, the LASSO-selected model included the downstream exon GC content and the RBP *RBM25*.

### 3.6 RBP binding features partially retain predictive power

The dedicated model trained exclusively on RBP-derived features achieved a mean AUC of 0.70±0.01 (Figure 2E), demonstrating that RBP binding patterns alone capture substantial information relevant to intron retention.

Interestingly, the corresponding scree plot (Figure 3C) reveals a notably different performance profile compared to previous models: the AUC reaches a plateau only after the inclusion of approximately 60 features. This slower saturation suggests that the RBP-mediated regulatory signal is not concentrated in a few dominant factors, but is rather distributed across a broad combinatorial network of protein-RNA interactions. Within this complex landscape, the RBP *MSI1* was identified as the top-ranked predictor (Table 2), highlighting its primary role in the model’s discriminative logic.

Within this predictor space, the LASSO analysis identified a broad set of regulatory protein signatures. The top-ranked features in the LASSO-selected model included *RBFOX2*, *RBM41*, *HNRNPD*, and *IGF2BP3*, followed by *SF1* and *ELAVL4* (Table 3). A high degree of consensus was observed between the Random Forest and LASSO approaches for this subset of features. Both methodologies consistently identified *MSI1*, *KHSRP*, and *ELAVL4* among the most discriminative predictors.

**FIGURE 5:**
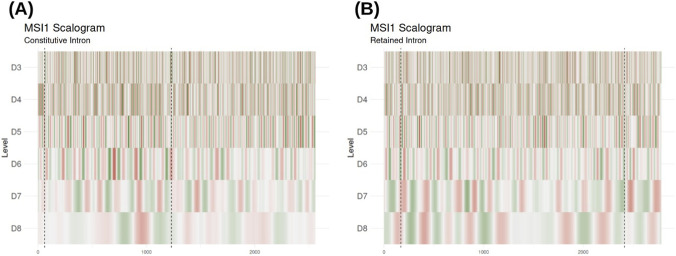
Wavelet multiresolution analysis of MSI1 binding signal within representative introns. **(A)** Profile of MSI1 binding density along a constitutively spliced intron. **(B)** Profile of MSI1 binding density along a retained intron. In both panels, the tracks (Level 3–6) show the wavelet coefficients at increasing scales of resolution. The color scale represents the wavelet coefficients: red indicates regions where the signal is in-phase with the wavelet basis, while green indicates regions in counter-phase. White/light gray areas denote low-magnitude coefficients, corresponding to low-affinity sequences or lack of significant structural motifs at that scale. Gray dashed vertical lines indicate exon-intron junctions.

To provide an interpretable illustration of the wavelet-based descriptors, we examined the spatial organization of MSI1 binding sites in a representative CI and RI (Figure 5). The CI profile (Figure 5A) is characterized by a fragmented and stochastic signal distribution across all scales. In particular, at higher levels of decomposition (D7–D8), the coefficients remain low in magnitude and lack spatial coordination, reflecting a scattered motif arrangement. In contrast, the RI profile (Figure 5B) displays a distinct multiscale architecture. While levels D3–D5 capture short-range binding events, the D7 and D8 levels reveal prominent, high-intensity regions that are consistently maintained along the intronic sequence. These features denote the presence of dense, organized clusters of MSI1 binding motifs that resonate at broader spatial scales, creating ‘regulatory hubs’ that are absent in the CI.

### 3.7 Repetitive elements features also partially retain predictive power

**FIGURE 6:**
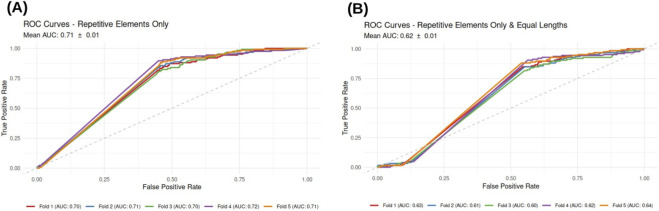
Performance of the models trained using only repetitive elements. **(A)** ROC curves showing the classification performance using all sequences (Mean AUC: 0.70±0.01 ). **(B)** ROC curves for the model trained on sequences with equal length distribution (Mean AUC: 0.62±0.01 ).

Finally, the dedicated model focused exclusively on Repetitive Elements-derived features yielded a mean AUC of 0.71±0.01 (Figure 6A). To assess whether this predictive power was independent of intron length, we re-evaluated the model using the length-matched dataset. Under these controlled conditions, performance decreased to a mean AUC of 0.62±0.01 (Figure 6B).

The corresponding scree plot (Figure 3D) reveals a sharp performance saturation, with only 7 features sufficient to capture the full predictive power of the model. These key predictors include members of the LTR, SINE, and LINE families, as well as Simple Repeats (Table 2), suggesting that specific classes of transposable elements and low-complexity sequences are non-randomly associated with the retention of specific introns.

The LASSO analysis also identified *LINE L1*, *LINE L2*, *SINE Alu* and *SINE MIR* as the most discriminative features (Table 3). The LASSO-selected features also included *LINE CR1*, which was not selected by the Random Forest model.

## 4 Discussion

This study provides a systematic, sequence-centric characterization of intron retention in the human genome and quantifies how multiple layers of sequence-encoded information jointly influence intron fate. By integrating structural features, splice site strength, repetitive element content, and multiscale RNA-binding protein (RBP) binding signatures within Random Forest and LASSO frameworks, we show that the predisposition to intron retention emerges from the combined weakening and simplification of regulatory signals rather than from any single dominant determinant.

Consistent with previous large-scale analyses (Braunschweig et al., 2014), the sequence length emerged as the strongest individual predictor of intron fate in our initial Random Forest model. However, our results demonstrate that while length carries high statistical power, it is insufficient to explain the underlying biological mechanisms of retention, due to its inherent correlation with other sequence-encoded features. The application of LASSO-based selection provided a critical framework for interpreting this dependency. When the model was constrained to penalize redundant variables, the global sequence length signal was largely replaced by more granular regulatory signatures, specifically the binding patterns of RBPs, along with the distribution of transposable elements, splice site strength, and GC content.

This shift indicates that the regulatory grammar of intron retention is not uniform but varies according to the physical scale of the intron. Specifically, we observe a transition from global determinants to localized regulatory signals: while the complete model is dominated by signatures such as MSI1 and KHSRP binding patterns, the removal of length bias prioritizes factors like ZNF326 and PPRC1. These differences highlight length-dependent regulatory patterns, suggesting that the ‘genomic space’ available in longer introns facilitates the accumulation of complex, multiscale signatures—such as the structured RBP binding and transposable element architectures—that are otherwise absent or less prominent in shorter sequences.

A central contribution of this work is the quantitative characterization of this organization through wavelet-based descriptors. Rather than focusing on simple motif counts, this approach captures the structural complexity of RBP binding signals along the sequence. We found that RIs consistently display reduced wavelet energy and lower sparsity across scales, reflecting a more uniform, less clustered, and overall less structured distribution of RBP binding sites compared to CIs. This reduced regulatory complexity—rather than mere length—may limit the efficient, localized recruitment of splicing factors, thereby biasing these introns away from co-transcriptional removal and establishing the foundational susceptibility for retention.

The prominent role of *MSI1* is particularly notable. Although *MSI1* is not generally considered a global splicing regulator, its extensive intronic binding landscape has been documented in glioblastoma cells (Uren et al., 2015). Most *MSI1* binding sites are located distal to splice junctions, suggesting a role beyond direct spliceosome recruitment. It has been proposed that *MSI1* may act as a scaffold for chromatin remodeling complexes such as *INO80C* and *SMARCD1*, thereby influencing the transcriptional and epigenetic context of splicing. In this framework, the structural complexity of *MSI1* binding in RIs may reflect a transcriptional environment that is less permissive to efficient constitutive splicing, rather than *MSI1* acting as a direct inducer of intron retention.

The *MSI1* mechanism aligns with the model proposed by Petrova et al. (2022), where sequence-encoded susceptibility is integrated with specific chromatin accessibility states and altered RNA Polymerase II kinetics. Furthermore, the ability of sequence-level features to act as determinants of the epigenetic landscape is consistent with the recent deep learning framework proposed by Daoud and Ben-Hur (2025), which demonstrates that IR-susceptible regions are characterized by specific transcription factor binding patterns and chromatin signatures that act together with the primary DNA sequence.

In addition to these regulatory signatures, the nucleotide composition—specifically the GC content—further modulates this landscape. Retained introns and their flanking exons are enriched in GC nucleotides, a property known to promote stable RNA secondary structures and influence RNA polymerase II elongation kinetics (Sznajder et al., 2018; Veloso et al., 2014). Both effects can perturb the temporal coordination between transcription and spliceosome recruitment, thereby favoring intron retention. Our results, particularly the feature importance from our models, indicate that GC content acts synergistically with the other features, rather than as an independent driver.

The intrinsic quality of core splicing signals remains a primary determinant of intron fate. Our results from the length-matched models consistently identified donor and acceptor splice site strengths as top-ranking features, a finding that was further corroborated by the LASSO selection. This suggests that the ‘intrinsic predisposition’ to retention is fundamentally rooted in a suboptimal recognition of the intron boundaries by the spliceosome.

Repetitive elements introduce an additional, non-trivial regulatory dimension. Contrary to observations in some plant systems (Li et al., 2014), retained introns in humans are significantly depleted of LINE and SINE elements, particularly Alu and MIR sequences. The predictive contribution of these elements in dedicated models suggests that specific repetitive architectures may facilitate constitutive splicing, possibly by introducing regulatory motifs, affecting local RNA structure, or modulating chromatin organization. This reflects a lineage-specific strategy: while TEs are abundant in both human and mouse, primate-specific Alu elements exhibit a unique propensity for exonization and the creation of novel intronic sequences—a process termed ‘intronization’ (Sela et al., 2007). Our results support the hypothesis that these lineage-specific retroelements have been uniquely co-opted to fine-tune transcriptomic complexity in humans.

Importantly, the density of repetitive elements exhibits a moderate correlation with intron length, reflecting the stochastic likelihood of transposable element insertion in larger genomic intervals. While the LASSO model prioritized specific repetitive families (e.g., SINE Alu and MIR and LINE L1, and L2) and assigned a null coefficient to the total length of the intron, this result should be interpreted with caution. Rather than implying that compositional quality independently outweighs intron length, it suggests that these specific transposable element families may effectively capture the regulatory information often associated with sequence extension. This interpretation is supported by our length-controlled analysis: when the repetitive elements-only model was trained on the length-matched dataset, its predictive performance decreased from an AUC of 0.71 to 0.62. This reduction confirms that a significant portion of the predictive signal provided by repetitive elements is coupled with intron length. Nevertheless, the remaining predictive power (AUC 0.62) indicates that these genomic patterns host intrinsic signatures that contribute to the predisposition for intron retention beyond a simple stochastic overlap with sequence length.

Notably, the model trained on length-matched datasets exhibited a reduction in predictive performance compared to the complete model. This drop suggests that, while sequence-level features are critical, they do not fully capture the information originally provided by the intron length. This performance gap likely reflects the fact that, in the absence of the ‘length proxy,’ the model requires additional layers of information to achieve high accuracy. Specifically, the ‘intrinsic predisposition’ encoded in the sequence may be necessary but not sufficient to determine the final splicing outcome in all contexts. As highlighted by Petrova et al. (2022), when sequence signals are suboptimal or ambiguous, the role of chromatin accessibility and nucleosome positioning becomes dominant. Therefore, the lower performance of the length-matched model may indicate that for many introns, the decision to be retained is highly dependent on the epigenetic and transcriptional context—such as the transcription factor networks identified by Daoud and Ben-Hur (2025)—which were not explicitly included as predictors in our sequence-centric analysis.

In this work, several limitations should be acknowledged. First, our analysis focuses on annotation-defined and coding transcripts retained introns and therefore captures intrinsic, sequence-encoded potential for retention rather than condition-specific or dynamic splicing responses observed in RNA-seq data. Second, RBP binding predictions are derived from PWMs and do not account for cellular context, protein abundance, or competitive binding. Finally, while our models achieve robust predictive performance, they do not explicitly model RNA secondary structure or epigenetic modifications, which likely interact with the features studied here.

Despite these limitations, this work establishes a scalable and interpretable computational framework for dissecting intron retention from primary sequence. By quantifying how multiple sequence-derived features jointly influence intron fate, it provides a principled basis for future studies of co- and post-transcriptional splicing regulation.

Although the proposed model achieves robust predictive performance (AUC = 0.86±0.02 ), several directions remain for future investigation. Extending this framework to other organisms will clarify whether the identified determinants represent conserved principles or species-specific architectures. In addition, retained introns may comprise heterogeneous subclasses driven by distinct regulatory mechanisms, motivating further stratification analyses. Finally, integrating dynamic regulatory layers such as transcriptional kinetics and epigenetic context will be essential to bridge static sequence features with co- and post-transcriptional splicing regulation.

## Data Availability

The original contributions presented in the study are included in the article/supplementary material, further inquiries can be directed to the corresponding author.
